# Structure/Function Analysis of Nonwoven Cotton Topsheet Fabrics: Multi-Fiber Blending Effects on Fluid Handling and Fabric Handle Mechanics

**DOI:** 10.3390/ma11112077

**Published:** 2018-10-24

**Authors:** Michael Easson, Judson Vincent Edwards, Ningtao Mao, Chris Carr, David Marshall, Jianguo Qu, Elena Graves, Michael Reynolds, Andres Villalpando, Brian Condon

**Affiliations:** 1 Southern Regional Research Center, New Orleans, LA 70124, USA; Vince.Edwards@ars.usda.gov (J.V.E.); Elena.Graves@ars.usda.gov (E.G.); Michael.Reynolds@ars.usda.gov (M.R.); Andres.Villalpando@ars.usda.gov (A.V.); Brian.Condon@ars.usda.gov (B.C.); 2 Performance Textiles and Clothing Research Group, University of Leeds, Leeds LS2 9JT, UK; N.Mao@leeds.ac.uk (N.M.); C.Carr@leeds.ac.uk (C.C.); D.Marshall@leeds.ac.uk (D.M.); J.Qu@leeds.ac.uk (J.Q.)

**Keywords:** fluid handling, fabric handle, greige cotton, nonwovens, topsheet, rewet, strikethrough, incontinence

## Abstract

Greige cotton (GC) has attracted interest in recent years as an eco-friendly, functional fiber for use in nonwoven topsheet materials. GC imparts favorable fluid management and sensorial properties associated with urinary liquid transport and indices related to comfort in wearable incontinence nonwovens. Nonwoven GC has material surface polarity, an ambient moisture content, and a lipid/polysaccharide matrix that imparts positive fluid mechanic properties applicable to incontinence management topsheet materials. However, a better understanding of the connection between functionality and compositional aspects of molecular, mechanical, and material property relations is still required to employ structure/function relations beyond a priori design. Thus, this study focuses on the relation of key indices of material fluid and sensorial functions to nonwoven topsheet composition. Greige cotton, polypropylene, bleached cotton, and polyester fiber blends were hydroentangled at 60, 80, and 100 bar. Greige cotton polypropylene and bleached cotton were blended at ratios to balance surface polarity, whereas low percentages of polyester were added to confer whiteness properties. Electrokinetic and contact angle measurements were obtained for the hydroentangled nonwovens to assess surface polarity in light of material composition. Notably, materials demonstrated a relation of hydrophobicity to swelling as determined electrokinetically by Δζ, ζ_plateau_, and contact angles greater than 90°. Subsequently, three blended nonwoven fabrics were selected to assess effects on fluid management properties including topsheet performance indices of rewet, strikethrough, and fluid handling (rate and efficiency of transport to the absorbent core). These materials aligned well with commercial topsheet fluid mechanics. Using the Leeds University Fabric Handle Evaluation System (LUFHES), the nonwovens were tested for total fabric hand. The results of the LUFHES measurements are discussed in light of fiber contributions. Fiber ratios were found to correlate well with improvement in softness, flexibility, and formability. This study provides insights that improves the understanding of the multifunctional properties accessible with greige cotton toward decisions valuable to selecting greige cotton as an environmentally friendly fiber for nonwoven topsheets.

## 1. Introduction

For decades, most incontinence topsheet manufacturers have used polypropylene (PP), which is hydrophobic and less expensive than greige cotton. Petroleum-based PP has been the material of choice in the incontinence topsheet market, with approximately 50 million tons produced annually [1], compared to cotton’s use, estimated to be approximately 2% (by volume/weight) of the total fiber consumption in nonwovens. Most of the cotton used, at present, in absorbent nonwovens is bleached [2]. However, given consumer demand for more environmentally friendly materials, there exists an opportunity for the increased usage of greige cotton fiber based on previously cited health and environmental issues [3]. Furthermore, the challenges for a PP fiber substitute are economical, process-related, and include properties that achieve optimal fluid handling while also improving on tactile properties. Additionally, it may be possible to reduce some of the environmental impact from PP by blending greige cotton fiber and achieve improved liquid handling and comfort.

**Figure 1:**
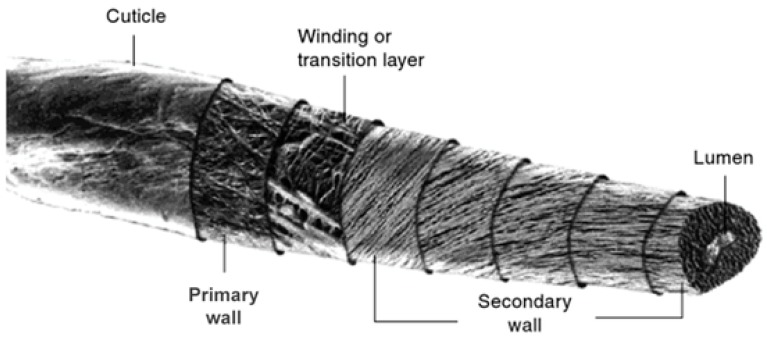
Macroscopic components of the cotton fiber.

The advantageous properties of the cotton may be described in terms of the fiber morphology and molecular composition. The primary cell wall and cuticle of the natural greige cotton fiber contain 1–2% pectins and waxes, as shown in Figure 1 [4]. During cotton fiber processing, pectins and waxes are removed by scouring at high temperature in the presence of caustic substances, exposing the secondary cell wall of the cotton fiber, which confers increased absorbency and hydrophilicity. On the other hand, when components of the fiber cuticle are not removed during nonwoven hydroentanglement, the greige cotton fibers retain the hydrophobicity associated with the cuticle waxes and are loosened, allowing an inlet of water to the hydrophilic cellulosic secondary cell wall. Thus, the resulting combination of hydrophobicity and hydrophilicity is a property useful for topsheet application.

The primary purpose of the absorbent topsheet is to facilitate the inlet of liquid to the acquisition and distribution layers, with rapid strikethrough and minimal rewet, providing efficient uptake of urine to the absorbent core as was first documented for disposable diapers [5]. The overall effect is to keep the skin dry and prevent dermatitis [6]. The topsheet is in direct contact with the user’s skin and, thus, fluid transports away from the skin with the absence of leakage (efficient fluid handling) that is paramount to topsheet performance [7]. PP topsheets have low water retention on the surface, but have good bulk fluid transport by way of treatment with surfactants to improve wetting behavior [8]. Given PP’s low cost and favorable fluid handling properties, it is understandable why it is the current material of choice for hygienic topsheets [9].

Another important goal of topsheet development is fabric comfort at the skin/topsheet interface. Various modelling methods involving both human subjects and instrumentation have examined the fabric–skin interaction in terms of friction (tribology), shear, and pressure for both woven and nonwovens [10]. Cottenden et al. [11] have described improved methodologies for modeling friction, pressure and shear at the skin–fabric interface with care sheets and incontinence pads. Studies have also been reported on skin–fabric interactions as a function of epidermal hydration on friction of human skin against textiles [12,13].

An individual’s evaluation of a piece of fabric often employs objective and subjective criteria that involve personal opinion and preference [14]. However, a fabric’s color is not considered a factor in handle determination. Although there are longstanding traditional and cultural associations between whiteness, purity, and cleanliness, these are separate issues from fabric handle. There are a few reports correlating fabric whiteness with fabric hand. For example, Yenket et al. concluded “that a pronounced visual effect, color, generally had little influence on the tactile hand sensory properties of fabrics using trained or consumer panelists” [15].

However, consumer preference for whiteness is accepted by textile manufacturers who tailor processes using oxidizing chemicals, optical brighteners, or bluing agents [16,17]. As mentioned earlier, these reagents have an impact on the environment through the use of water, energy, and the production of hazardous waste streams. Thus, the present research has chosen to blend blue polyester fibers to address this issue alongside functional performance properties.

Over the past several decades, a few methods have been developed to quantify human touch perception. Tactile perception is a complex interplay of rubbing, squeezing, and stretching that involves a multidimensional analysis of the fabric. The conclusions from instrumental evaluation methods, such as the Kawabata Evaluation System for Fabrics (KES-F) and Fabric Assurance by Simple Testing (FAST), are not necessarily objective because of their dependency on statistical data [18], artificial neural network outputs [19], and relationships between measured fabric mechanical properties and the subjective hand preferences of human panels using reference fabrics [20].

Furthermore, the Kawabata method examines fabric hand in a limited single dimensional approach, and the standard fabrics used are solely based on 100% woven wool fabrics. In a handle assessment method based on extraction [13,21], a fabric is forced through a narrow opening, such as a ring or nozzle, creating deformations in multiple directions similar to that occurring during human hand interactions. However, the resultant quantitative assessment of fabric handle is based upon statistical principal component analysis and the conclusions of subjective assessment. Additionally, the random fabric deformations that occur in the extraction systems can lead to problems of reproducibility. In the Fabric Touch Tester [22], tactile comfort is evaluated based upon multiple fabric bending deformations, rather than the fabric responses during buckling deformation. Existing approaches outlined here do not quantify the recovery of the fabric following initial deformation, which is known to affect the tactile perception in subjective fabric hand evaluations [23].

**Figure 2:**
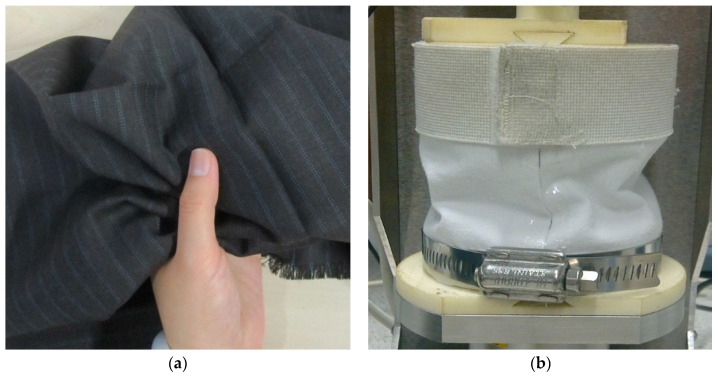
Compressive fabric deformations, (**a**) by human hand and (**b**) by Leeds University Fabric Handle Evaluation System (LUFHES) objective handle method. Ref: http://www.innovationintextiles.com/objectively-evaluating-fabric-handle/.

Assessment of tactile comfort at the skin–fabric interface strives to improve the objective and subjective evaluations of fabric handle. The criteria for evaluating clothing comfort of woven and nonwoven fabrics has been reviewed [24], with the goal of bridging the divide between objective and subjective handle evaluations. This is at the heart of the fabric handle evaluation system that has been developed by the Leeds University Fabric Handle Evaluation System (LUFHES) [25]. This system mimics the multidimensional rubbing, squeezing, and stretching human interactions that play a role in handle determination. Figure 2b depicts the LUFHES method in operation, which demonstrates the compression buckling of a fabric cylindrical shell. Fabric handle indices are represented by using the different types of fabric deformations produced, which are quantified by the energy consumed during the cyclic deformation/recovery process.

Previously, research identified some of the fluid handling and fabric handle indices that are required for functional incontinence topsheets in hydroentangled greige cotton [26]. However, the structure/function relationship has not been elucidated sufficiently to predict cotton fiber substitution effects in topsheet design. Moreover, this needs to be done in light of recent results that also document the utilization of blue polyester fiber to improve nonwoven whiteness [27]. Thus, this paper addresses intimate blending and hydroentangling cotton fibers with polypropylene, bleached cotton, and polyester fibers to form nonwoven fabrics, which are further characterized at a molecular/material level for their electrokinetic and contact angle profiles. Therefore, molecular surface properties are linked to fabric hand and fluid management mechanics, with the goal of designing and developing incontinence products with robust fluid handling and improved fabric hand.

## 2. Materials and Methods

Greige cotton fibers (True Cotton™) were obtained from T. J. Beall of Greenwood, Mississippi. These cotton fibers were precleared using a proprietary process which removed 99.99% of all impurities (field trash, stems, leaves, etc.) in a nonaqueous, mechanical process (http://www.tjbeall.com/natural-fibers-nonwoven/true-cotton). Blue polyester fibers were supplied by Palmetto Synthetics LLC of Kingstree, South Carolina. Bleached cotton fibers were produced from a bale of True Cotton™ that were bleached by Tintoria Piana US in Cartersville, Georgia. The polypropylene fibers were purchased from Fibervisions^®^ of Duluth, Georgia. All fibers were received and used without further modifications.

All nonwoven fabrics were prepared at the USDA Southern Research Center in New Orleans, Louisiana. The manufacturing specifics are as follows: staple fibers were weighed and hand-blended, and then opened on an Opening and Blending Line, consisting of a fiber hopper, Hollingsworth WR cleaner, D106 distributor, and 310 Fine Opener and another D106 distributor. All fiber blends were processed through two times, to achieve optimal openness and blending.

The fiber blends were chute fed to a 40 inch-wide textile card fitted with 4 Cardmaster plates (Saco Lowell), followed by feeding the card web into a commercial crosslapper and needlepunch (NP) machine (Technoplants s.r.l., Pistoia, Italy). The NP processing parameters were varied to achieve the target weights (25–35 g/m^2^). The NP fabrics were converted into hydroentangled (HE) nonwoven fabrics on a 1 m wide Fleissner pilot-scale hydroentanglement system (Trützschler Nonwovens GmbH, Dülmen, Germany) running at a constant production speed of 5 m min^−1^. The hydroentanglement (HE) system utilized two pressure heads: one low pressure for fabric wet-out maintained at a constant pressure of 30 bar during fabric production; and the second is a high-pressure head maintained at 60, 80, and 100 bar during separate fabric production runs. The final bonding nozzle is suspended over a 24 mesh embossing drum to cast a 24 mesh pattern into the nonwoven material. Each strip on the bonding pressure heads consisted of 50 orifices per inch, with an orifice pore size of 120 µm. The water used for the HE fabric production was ambient temperature, which was approximately 25 °C. Following HE, the fabrics were fed directly through a gas-fired fabric-drying oven (Trützschler Nonwovens GmbH) at ~160–180 °C (depending on fiber melt points) and wound into rolls.

The hydroentangled fiber blend samples were analyzed for spectroscopic properties using a Konica Minolta CR-410 chromameter. The chromameter analyzed samples for color space properties in terms of CIELAB color coordinates (L*, a* and b*) using the 2° observer and the CIE standard illuminant C. L* represents lightness and can be measured independently of color hue. A decrease in lightness is associated with a decrease in fabric reflectance. Normal to the L* axis (lightness) are the +b* to −b* axis and the perpendicular +a* to −a* axis, where b* represents the color yellow (90°), −b* blue (270°), +a* red (360°), and −a* green (180°). Each recorded L*a*b* measurement was the average of three reflectance readings, obtained by rotating the samples after each measurement.

The contact angle of a water drop on nonwoven fabrics was measured using a VCA Optima XE (AST Products, Inc., Billerica, MA, USA). Runs were performed with a 5 µL drop of distilled water syringed onto the fabric. The image of the drop was immediately captured and analyzed to yield a contact angle. Contact angles on twelve different areas were measured, and their average value was recorded.

The determination of the ζ-potential was carried out with the Electro Kinetic Analyzer (Anton Paar, Ashland Va.) using the Cylindrical Cell developed for the measurement of fibrous samples. When a fiber absorbs liquid and swells, the surface charges become farther separated, and the absolute value of its ζ-potential decreases. Two kinds of measurements were made on each sample: (1) swell tests to measure the rate and extent of fiber swelling (at a given pH) and (2) a pH titration in which the swelling is measured as a function of pH. All ζ-potential measurements were made in a 1 mM KCl electrolyte.

In the electrokinetic apparatus, the streaming potential is measured, and the zeta potential determined from the Smoluchowski equation [28,29]:

ζ = dU/dp × ηκ/(ε_γ_ ε_0_), (1)

where U is the streaming potential—the potential generated when an electrolyte is forced to flow over a stationary charged surface, p the pressure, ε_γ_ and ε_0_ the dielectric constant and the vacuum permittivity, η the viscosity, and κ is the conductivity of the measuring fluid. Surface conductivity of the fibrous samples was not taken into account. pH titrations were performed over a pH range of 11.0 to 2.5 to ensure recording both the isoelectric point (IEP) and the plateau potential. (The IEP is the pH at which ζ = zero, and provides insights into the surface association/dissociation processes.)

Fabric handle was evaluated using the Leeds University Fabric Handle Evaluation System (LUFHES). Six nonwoven fabric specimens, three in the cross direction (CD) and three in the machine direction (MD), were tested. Cyclic compression buckling of 30%, cyclic shear deformation of 5° twisting, and fabric–fabric self-friction properties of the six nonwoven fabrics were evaluated during the biaxial deformations of the fabrics. Six fabric handle descriptors and indices are defined based upon the energy consumed either to deform a fabric or to recover the deformation from the deformed state, as defined below.

Fabric sponginess (SP): the extent to which a fabric spontaneously recovers from deformation when the external deformation force is withdrawn (elasticity). Fabric crispness (CR): the level of unrecoverable permanent deformation produced under external deformation force. Fabric flexibility (FL): the resistance to recovery of the original dimensions after deformation by an external force. Fabric stiffness (ST): the resistance to deformation by an external force. Fabric softness (SF): the combined effect of the resistance to deformation and the resistance to the recovery subject to an external force. The total fabric handle value (TFHV) is defined as

TFHV = SP + CR + FL + ST.
(2)

Thus, the greater the TFHV value, the greater amount of energy is required to deform the fabric and to recover from the fabric deformation. An additional fabric handle parameter, referred to as fabric smoothness (SN), is defined as the dynamic fabric-to-fabric friction coefficient, or the coefficient of dynamic friction between the fabric and itself. In addition, fabric formability (30b), which is defined to represent a fabric’s ability to be conformed to a 3D shape, could be calculated from the fabric compression buckling force measured in the tests.

Liquid acquisition, strikethrough, and rewet tests were performed by Marketing Technology Service, Inc. Kalamazoo, Michigan. According to EDANA/INDA WSP 70.3 and WSP 70.7, the liquid strikethrough time involves determining the time taken for a known volume of test liquid (simulated urine) applied to the surface of a test piece of nonwoven topsheet, which is in contact with underlying standard absorbent pads to pass through the fabric. Rewet testing was performed according to EDANA/INDA WSP 80.10 and WSP 70.8. In this procedure, the wet back of synthetic urine through a prepared sample onto a filter paper is determined. After applying a defined volume of liquid upon the prepared sample (strikethrough time test), a simulated baby weight is applied onto the specimen at a predefined speed and dwell time. Using a specific filter paper and an electronic balance, the amount of liquid that transfers back through the specimen’s surface into the filter paper is determined. The liquid acquisition test evaluates in real time how effectively liquids penetrate into the absorbent core of the absorbent device at any flow rate. A 0.9% saline solution is applied to the specimen at a specific volume and flow rate. The dynamic change of the total volume of fluid acquisition and overflow liquids over the duration of the testing was obtained.

## 3. Results

### 3.1. Design and Selection of Fabric Compositions

**Figure 3:**
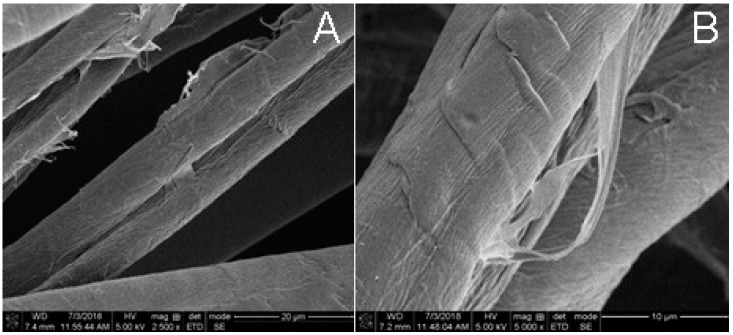
Samples 2 (image (**A**)) and 4 (image (**B**)) showing fibrillation and the partial separation of cotton fiber cuticle wax as a result of hydroentanglement.

At the outset of the study, ten nonwoven fabrics (Table 1) were designed and prepared based on previous findings outlining the material surface polarity, liquid handling, fabric hand, and whiteness [26], with the goal of demonstrating the cooperative fluid handling, fabric hand, and whiteness profiles that greige cotton and polypropylene combinations exercise. Hydroentanglement of the blends with greige cotton confers combined hydrophobicity and hydrophilicity to the material through exposure of the cellulosic cell wall and loosening of the fiber cuticle waxes, as depicted in the SEM in Figure 3. Polypropylene and polyester also contribute hydrophobicity to the material’s polar surface properties. Bleached cotton is included in the ratio blends contributing hydrophilicity.

### 3.2. Fabric Whiteness

Greater hydroentanglement pressures more effectively removed the yellow surface waxes on greige cotton fibers than lower hydroentanglement pressures. There is also a process-related reduction of L* (lightness), from a control high of 94.29 to a low in sample 10 (97% greige cotton) of 85.41 when hydroentanglement pressure was applied at 100 bar versus 60 bar, respectively as shown in Table 1. This trend, related to the effect of hydroentanglement pressure on yellowness, is especially evident in samples which contain increasing percentages of greige cotton. Similarly, as the percentage of greige cotton increased, the b* measurement also increases.

It has been previously reported by Easson et al. that small percentages of blue fiber can give the appearance of increased whiteness using the principles of additive color mixing [27] without the need for chemical agents. Simply stated, when blue and yellow fibers are intimately blended, the human eye cannot distinguish them. The two colored fibers are perceived as white, which is consistent with the colorimetry measurements which categorize the elements of reflective light responsible for the visual effect. This same principle is behind projection televisions which blend red, green, and blue lights to form picture images [30]. Thus, as seen with the samples containing blue polyester fibers, whiteness is increased.

### 3.3. Electrokinetic and Contact Angle Analysis in View of Topsheet Layer Function

Shown in Table 2 are the results of the zeta potential titrations for the ten nonwoven blends hydroentangled at 60 bar. The ζ_plateau_, isoelectric point, Δζ, swell ratios, and rate of swelling (k) have been previously employed to characterize compatible material interfaces of topsheets (TS) and acquisition distribution layer (ADL) materials found in marketed incontinence products [31].

**Figure 4:**
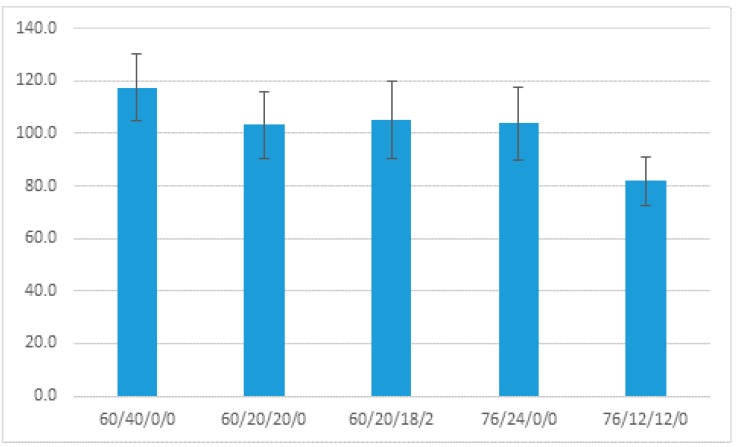
Contact angle determination for five fabric blends. Numbers in x-axis represent percentages of greige cotton, polypropylene, bleached cotton, and blue polyester fibers.

As shown previously for cotton, the swelling of the material, as measured by the Δζ, is a function of the materials’ expansion within the ion shear plane during pH titration [32,33,34]. Thus, based on polarity, porosity, percent moisture content, and the interaction of hydrophilic and hydrophobic fibers, a material will expand to some extent while undergoing zeta potential titration. The ζ_plateau_ values for the ten blends range from −34 to −25 mv, and the influence of the hydrophobicity of polypropylene is evident, as the blends containing a larger percentage of polypropylene have the more negative zeta potential. This is consistent with the more hydrophobic contact angle values shown in Figure 4.

Contact angle testing confirmed a finding first reported by Sawhney et al. and identified in GC topsheet [2,26], demonstrating that higher HE pressures remove the cotton cuticle waxes and increase the hydrophilicity of the material surface. However, seven of the ten nonwovens manufactured at 60 bar had measurable contact angles characteristic of a hydrophobic fabric as shown in Figure 4, and this correlates with a ratio of polypropylene in the blend that is greater than 12%. It is noteworthy that when polypropylene in fabrics with greige content of 60% and 76% is decreased by fifty percent of the fiber composition in the fabric, the contact angle is reduced from 118° to around 105°, and from 105° to 82°, respectively.

### 3.4. Analysis of the Fabric Tactile Properties

**Figure 5:**
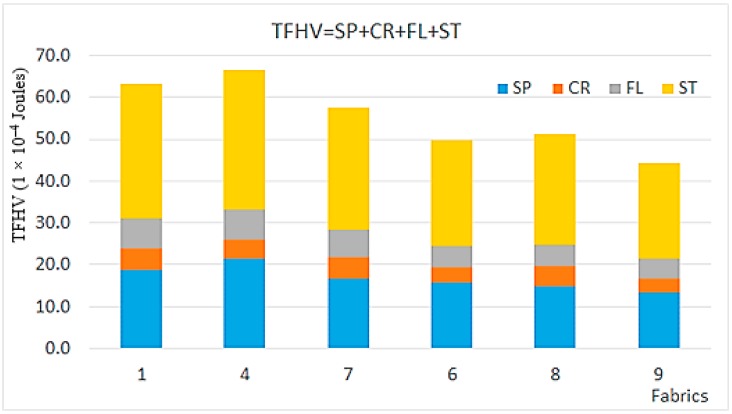
Comparison of total fabric handle value (TFHV) of the six nonwoven fabrics.

The results of the LUFHES tactile descriptor valuation are shown in Table 3. A comparison of total fabric handle values (TFHV) of the six nonwovens examined in this study is shown in Figure 5. From a structural standpoint, the blends analyzed for fabric handle indices may be grouped into three classifications: (1) samples 1 and 4 contain a higher percentage of polypropylene (PP) fibers (about 24–40%) relative to the greige cotton (60–76%); (2) Fabric 7 has a greater proportion of greige cotton fibers (90%) and relatively less PP fiber (10%); (3) In the third group (6, 8, 9), all the hydroentangled fabrics contain a relatively smaller proportion of polypropylene (PP) fibers (about 10–12%) and a relatively large proportion of greige cotton fibers (76–90%), with the presence of bleached cotton (3–10%) and very low levels (0–2%) of blue polyester fibers.

#### 3.4.1. Softness

The two fabrics in the first group containing a greater proportion of PP fibers (about 24%–40%) are the least soft fabrics (SF = 4.0–4.1 × 10^−5^ J). By contrast, Fabric 7, containing the greatest proportion of greige cotton (90%), is relatively softer (SF = 3.6 × 10^−3^ J). Fabrics 6 and 8, in the third group, contain the smallest amounts of PP fiber (5% in the fabric 8) or contain a small proportion of blue polyester fibers (2%) and are even softer (SF = 3.1–3.2 × 10^−3^ J). However, Fabric 9, which contains both the smallest proportion of PP fibers (5%) and a small proportion of blue polyester fibers (0%–2%), is the softest fabric of the six tested (SF = 2.8 × 10^−3^ J).

#### 3.4.2. Stiffness, Sponginess, and Total Fabric Handle Value (TFHV)

Based on considerations of the relative contributions of greige cotton and polypropylene fabric, 4 has the greatest sponginess (SP = 2.1 × 10^−3^ J) and the greatest TFHV (TFHV = 6.8 × 10^−3^ J). On the other hand, fabric 9 (90% greige cotton) has the smallest sponginess (SP= 1.3 × 10^−3^ J) and TFHV (TFHV = 4.5 x 10^-3^ J). Fabrics 1, 4, and 7 have relatively great flexibility value (FL = 6.7–7.4 × 10^−4^ J) and, notably, do not contain bleached cotton. However, fabrics 6, 8, and 9 have relatively smaller flexibility value (FL = 4.7–5.0 × 10^−4^ J), and 9 has the greatest flexibility (or the lowest flexibility value FL = 4.7 × 10^−4^ J).

#### 3.4.3. Crispiness and Smoothness

The fabrics 1, 4, 7, and 8 have relatively greater crispiness (CR = 4.6–5.1 × 10^−4^ J) and, in contrast, fabrics 6 and 9, which have similar values, have smaller crispiness (CR = 3.4–3.7 × 10^−4^ J). This is consistent with fabrics 6 and 9 containing 2% of the blue polyester fibers.

There are some trends in fabric smoothness. Fabrics 4 and 9 have the smoothest surface (SN = 0.675 and 0.730, respectively). This is consistent with fabric 9 being the softest fabric and fabric 4 containing a greater proportion of PP fibers (24%), as they might link to the formation of surface ribs/grooves formed during hydroentanglement process; both of them might have formed a smoother surface during the hydroentanglement process. Fabrics 1 and 6 have medium smoothness (SN = 0.902 and 0.841, respectively) consistent with a greater proportion of PP fibers (24% and 12% respectively) and the presence of a small amount of polyester. Fabric 7 and 8 have the least fabric smoothness (SN = 0.968 and 0.996 respectively), and this is consistent with these fabrics containing the greatest proportion of greige cotton fibers (90%), and polyester fibers that are absent.

#### 3.4.4. Formability

The fabrics 6 and 9 have the greatest formability (FMR = 0.35 and 0.37 mm^2^, respectively). Both fabrics contain polyester, and are principally composed of greige cotton fibers (86% for the fabric 6 and 93% for the fabric 9). Fabrics 4 and 7 have medium formability (FMR = 0.32 mm^2^), they contain a lower proportion of cotton fibers (76% and 90% of cotton fibers, respectively) in comparison with fabrics 6 and 9. Fabrics 1 and 8 have the smallest formability (FMR = 0.27 and 0.29 mm^2^, respectively), and they are, coincidently, the fabrics containing smallest (60%) and greatest (90%) proportion of cotton fibers, respectively, without the presence of polyester.

### 3.5. Strikethrough, Rewet, and Fluid Acquisition of Penetrant-Treated Greige Cotton Fabrics

**Figure 6:**
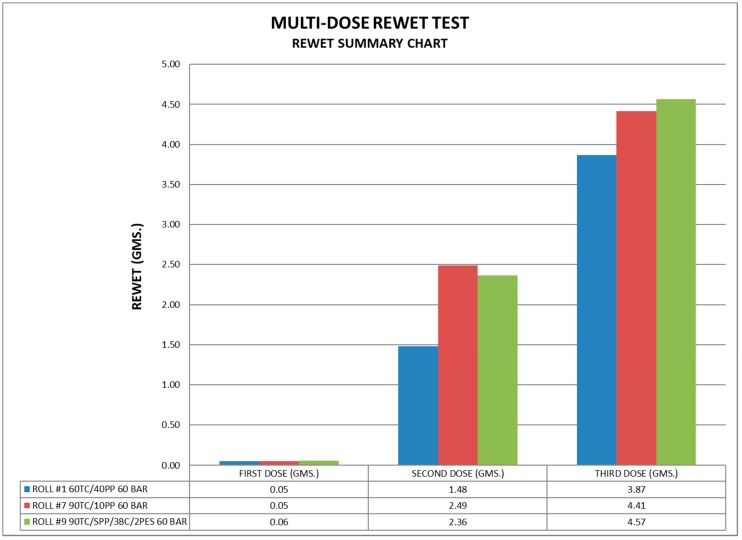
Comparison of multi-dose rewet of three nonwoven fabrics.

A group of three samples was selected to evaluate functional liquid handling properties central to incontinence management. In contrast with previously reported studies where the functional performance of 100 percent greige cotton was demonstrated, a ratio of greige cotton to polypropylene in the bi-component fiber blends was compared with a blend containing mostly greige cotton with lower percentages of polypropylene, bleached cotton, and polyester [26]. As shown in Figure 6, the rewet of the bi-component blend, with a ratio of 60:40 GC/PP, performed significantly better than the other two containing 90 percent GC.

**Figure 7:**
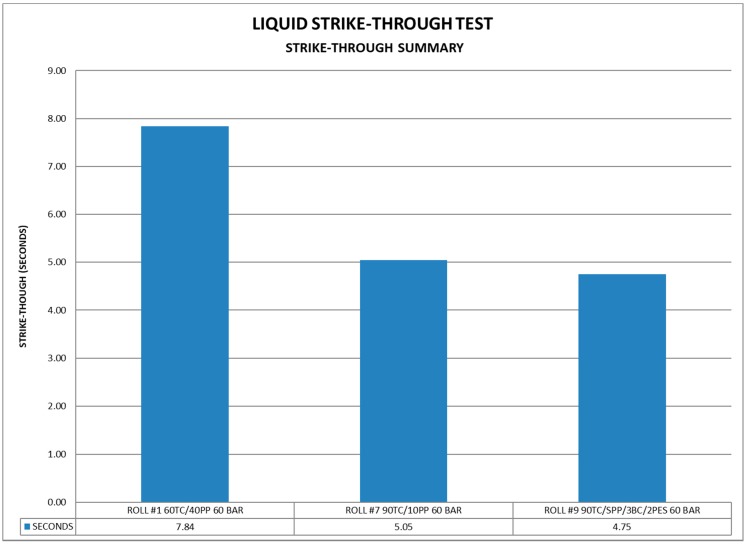
Strikethrough values for Samples 1, 7, 9.

As shown in Figure 7, it is apparent that a higher percentage of greige cotton facilitates improved strikethrough, since an approximate 40 percent increase in the rate of liquid uptake was observed between samples 1 (60:40, GC/PP) and 7 (90:10, GC/PP).

**Figure 8:**
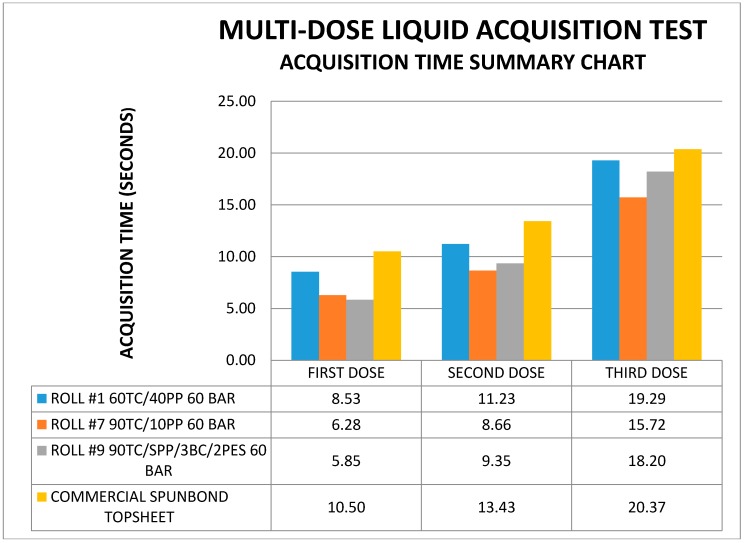
Comparison of liquid acquisition of three nonwoven fabrics and a commercial source.

As shown in Figure 8, the rate of overflow and leakage were as much as 50 percent improved in the 90 percent greige cotton blend at the first insult of 20 mL. In the second insult, the leakage and overflow differences were decreased to 25 percent, which was still retained in the third insult in composition 7.

## 4. Discussion

At the outset of the study, ten nonwoven fabrics were designed and prepared, based on previous findings outlining the material surface polarity, liquid handling, fabric hand, and whiteness [26], with the goal of demonstrating the cooperative fluid handling, fabric hand, and whiteness profiles that greige cotton and polypropylene combinations exercise. Hydroentanglement of the blends with greige cotton confers combined hydrophobicity and hydrophilicity to the material through exposure of the cellulosic cell wall and loosening of the fiber cuticle waxes, as depicted in the SEM in Figure 3. Polypropylene and polyester also contribute hydrophobicity to the material’s polar surface properties. Bleached cotton is included in the ratio blends contributing hydrophilicity.

Greater hydroentanglement pressures more effectively removed the yellow surface waxes on greige cotton fibers than lower hydroentanglement pressures. There is also a process-related reduction of L* (lightness), from a control high of 94.29, to a low in sample 10 (97% greige cotton) of 85.41, as hydroentanglement pressures decreased from 100 bar to 60 bar. This trend related to the effect of hydroentanglement pressure on yellowness is especially evident in samples which contain increasing percentages of greige cotton. Similarly, as the percentage of greige cotton increased, the b* measurement also increases.

In 2017 Easson et al. reported that small percentages of blue fiber can give the appearance of increased whiteness using the principles of additive color mixing [27] without the need for chemical agents. When intimately blended with light yellow greige cotton, certain shades of blue fibers cannot be distinguished from the yellow fibers by the human eye. The two colored fibers are perceived as white, which is consistent with the colorimetry measurements which categorize the elements of reflective light responsible for the visual effect. This same principle is behind projection televisions which blend red, green, and blue lights to form picture images [30]. Thus, as seen with the samples containing blue polyester fibers, whiteness is increased.

Considerations of material surface polarity are central to incontinence topsheet (TS) and acquisition distribution layer design (ADL) [31]. The use of electrokinetic measurements to demonstrate the relative contributions of TS and ADL to functional design has previously been demonstrated for heavy to light incontinence materials, and the ability to use greige cotton to modulate functional performance has been reported [26,31].

The material zeta potential (ζ-potential) is characterized through pH titration correlated to change in charge (millivolt increments), and ζ_plateau_ is the point where charge equilibrium is attained on the material surface. Zeta plateau (ζ_plateau_) can be either positive or negative, depending on its surface chemistry. Zeta potential analysis is highly applicable to absorbent materials, and provides a tool useful in the analysis of fluid dynamics in aqueous systems based on electrochemical double layer model [28].

The electrochemical double layer model has parallel properties to the fluid transport that occurs at the solid–liquid interface of incontinence material layers, i.e., there is transport of fluid through a material with a certain surface polarity that results in the swelling of a polar, porous material. Thus, zeta potential decrease (a change in the electrostatic potential at the shear plane of the electrochemical double layer) is caused by swelling of the fibers and outward movement of the aqueous shear plane where ions are in contact with the outer Helmholtz ion plane on the fiber surface [28,29]. An increase in swelling of the material occurs as the shear plane moves out to the more diffuse layer of ions surrounding the surface causing a decrease in ζ-potential.

The indication of a hydrophobic surface on greige cotton nonwovens, shown here, is consistent with incontinence topsheet functionality [26,33]. In addition, the zeta potential values are within an acceptable range, based on previously reported values for the TS of incontinence underwear and moderate or light incontinence products that contain polypropylene, cellulose, and polyester [31]. On the other hand, the Δζ values are within an acceptable range (0.019–0.15) for TS employed in incontinence underwear, moderate incontinence liners, and bed pads that contain polypropylene, cellulose, and modified acrylic acid [31]. As previously demonstrated, the degree of swelling of the TS and ADL in an incontinence product plays a role in designing functional performance parameters, such as strikethrough, rewet, and fluid transport to the absorbent core [33]. Rate of swelling (k) varied from 0.021 to 0.001 min^−1^, and was more accelerated in materials containing higher levels of bleached cotton (greater than 3%). Rate of swelling is also mediated by the amphiphilic surface polarity of the material, i.e., a combination of bleached cotton, greige cotton, and polypropylene.

Fabric handle analysis using the Leeds University Fabric Handle Evaluation System (LUFHES) method was limited to six samples representative of a range of surface polarity. As stated earlier, the greater the TFHV value derived in the LUFHES, the more energy that is required to deform the fabric and to recover the deformed fabric. Thus, a lower TFHV value is more desirable for a softer fabric. The results of the LUFHES tactile descriptor valuation are shown in Table 3.

Table 3 indicates that the standard deviation of the TFHV of these six fabrics were in a relatively large range (between 7% and 11% of their average TFHV). Such greater deviation was mainly due to the well-known anisotropy of hydroentangled nonwoven fabric properties in both machine direction (MD) and cross direction (CD) [34,35]. It is recognized that nonwoven fabrics have inherent anisotropic, and non-uniform properties in different directions. Thus, there is a significant difference between the average values of each index in different directions (i.e., frequently, the average value of each index in MD is about 1.2~1.6 times that in CD). The great difference of the objective measurement results in a different orientation in which a fabric may be positioned. This aspect of the assay may also be inherently one of the diverse subjective evaluation results among different evaluators in a human evaluation panel, and is consistent with the difference of the objective measurement results in different directions of a fabric, leading to the diverse subjective evaluation result in human evaluation panels [14,20].

Hydroentangled fabrics containing more greige cotton fibers and a small proportion of blue polyester fibers yield a softer fabric than those with higher PP. It is noticed that fabric 1, containing least greige cotton, is not the least soft fabric, especially in comparison with fabric 4. This is possibly due to the reason that polypropylene fibers have a relatively greater bending rigidity than cotton fibers, and might need greater energy in the hydroentanglement process to form fiber tanglement; thus, fabric 1, which has a higher proportion of PP fibers (40%), might have smaller hydroentanglement intensity (HI) (34b) than fabric 4, which has a smaller proportion of PP fibers (24%) in the level of the water jet pressure applied; smaller hydroentanglement intensity (HI) would lead to a relatively loose and bulky fabric, which should be relatively softer (34b). Previously, it was shown that a 50/50 greige cotton/polypropylene blend had a TFHV that is five times less than a commercial spunbond nonwoven, and this is predominantly due to higher softness and more sponginess, flexibility, and crispiness (18). A similar trend is also found in this study with the fabric stiffness (ST), sponginess (SP), and TFHV. For example, lower amounts of both polypropylene and bleached cotton tend to improve fabric THFV and flexibility when the fabric is predominantly composed of greige cotton.

Fabric formability (FMR) represents a fabric’s ability to be conformed to a 3D shape. Thus, a greater FMR value represents the fabric’s greater ability to be conformed to a 3D shape. The relative order of formability observed in this study is consistent with the presence of blue polyester fibers in those fabrics. This suggests the potential to utilize polyester to enable greater formability. Moreover, in the absence of polyester fibers, it is possible that either too small (60%) or too high (90%) amounts of cotton fibers could lead to smaller fabric formability, and fabrics containing a suitable proportion (76–90%) of greige cotton fibers enable a relatively greater formability. This suggests that cuticle waxes from greige cotton may increase formability. This finding also suggests that more malleable hydrophobic materials, such as wax and polyester, may improve fabric formability.

Optimal strikethrough, rewet, and fluid acquisition properties in an incontinence topsheet are essential for modulating levels of incontinent episodes in light, moderate, and heavy capacity categories [31]. As shown in Figure 6, the first rewet insult demonstrated sufficiently low rewet for all three samples, consistent with being undetectable by the skin [36]. On the other hand, the rewet values resulting from the second liquid insult to the material was within the margins of error of rewet previously reported for 100 percent GC, and twice the rewet capacity of a commercial spunbond sample [26]. The significant difference found in the rewet values between 60 percent greige cotton and 90 percent greige cotton is consistent with a higher ∆ζ and swell ratio found in the electrokinetic profile of nonwoven fabric 1 versus fabrics 7 and 9 (Table 2). Swell ratio has previously been correlated with liquid gradient and polar surface functionality between and within incontinence product layers i.e., topsheet, acquisition, and distribution layers [31]. In the context of topsheet rewet functionality, improvement in rewet is a function of interfacial liquid uptake and retention between the topsheet prototype material (60:40, GC/PP) and the absorbent core. Moreover, a larger swell ratio in fabric 1 suggests a greater expansion of the hydrophobic channels and interstitial spaces distributed within the test sample, and between the sample and absorbent core as pressure (2.5 kPa) simulating body weight is exerted, and a capacity to retain more liquid. This is also interesting in light of previous determinations showing that polypropylene swells at a greater rate than greige cotton [33]. However, this may be due to its lower density and porous nature. In this study, materials with less polypropylene swell more. Thus, the polar gradient formed between hydrophilic and hydrophobic portions of the material work synergistically to improve absorbency and retention of liquid by increasing the web interstitial space and modulating pore size [37,38].

In contrast with rewet functionality found in the topsheet, designing optimal strikethrough is largely dependent on accelerating both the wicking and retention capacity of the material. Strikethrough properties are, in part, optimized in commercial polypropylene samples by the application of surfactants [8] to promote rapid inlet of urine. However, the strikethrough values were somewhat attenuated compared with previously reported ones for greige cotton, due to the absence of surfactant treatment. The improved strikethrough observed with the 90 percent greige cotton is consistent with the structure of greige cotton which, as discussed previously, has some amphiphilic properties characteristic of surfactants i.e., molecules with a polar and nonpolar face [33]. Moreover, the distribution of bleached cotton on the surface of the material would be expected to increase wicking.

One of the most interesting results of the fluid handling experiments was the improvement in liquid acquisition observed over commercial spunbond topsheet performance in the multi-dose liquid acquisition test. Thus, in contrast with previous studies where 100 percent greige cotton was comparable to spunbond polypropylene topsheets, it is evident, here, that GC/PP blends improve liquid acquisition properties over commercial spunbond polypropylene topsheets.

## 5. Conclusions

Greige cotton, polypropylene, bleached cotton, and polyester fibers, were blended in discrete ratios and examined for whiteness, tactile, and fluid handling properties for topsheet application. In Leeds University Fabric Handle Evaluation System (LUFHES) tactile analyses, nonwoven blends containing low percentages of polyester and polypropylene and high percentages of greige cotton and bleached cotton were shown to have the lowest total fabric handle value (TFHV). Fiber ratios demonstrated a relation of hydrophobicity to swelling, as determined electrokinetically by isoelectric point (Δζ, ζ_plateau_, and contact angles greater than 90°. The influence of the hydrophobicity of polypropylene is evident as the blends containing a larger percentage of polypropylene have the more negative zeta potential. Seven of the ten nonwovens manufactured at 60 bar, with a ratio of polypropylene in the blend greater than 12%, had measurable contact angles greater than 90 degrees and are hydrophobic, which is consistent with incontinence topsheet functionality. The rate of swelling (k) was greater in nonwovens containing a greater amount of bleached cotton and a blend of amphiphilic fibers. Blue polyester fiber did improve fabric whiteness and factored in favorably in handle determination, but whiteness itself was not a factor in handle. On the other hand, improved liquid acquisition may work synergistically with improved formability, as observed with fluid management and fabric handle, respectively. The mechanics of fabric hand and fluid management functioning synergistically could improve incontinence material leakage and promote a more efficient retention of fluid in the absorbent core. The analyses of three nonwoven fabrics for rewet, strikethrough, and fluid handling aligned well with commercial topsheet fluid mechanics. Finally, this research provides valuable insights into nonwoven topsheet material selection, and provides a basis for better understanding of the fluid and hand dynamics that greige cotton imparts for incontinence applications.

## Figures and Tables

**Table 1:** Composition and L*a*b* Whiteness Measurement of Hydroentangled Nonwoven Rolls.

Sample	Component 1	Component 2	Component 3	Component 4	(bar)	L*	a*	b*
	Greige Cotton	Polypropylene	Bleached Cotton	Blue Fiber				
	% (grams)	% (grams)	% (grams)	% (grams)				
1	60 (2722)	40 (1814)	0	0	100	87.68	−0.45	5.9
	60 (2722)	40 (1814)	0	0	80	89.53	−0.41	7.6
	60 (2722)	40 (1814)	0	0	60	90.01	−0.26	8.94
2	60 (2722)	20 (907)	20 (907)	0	100	89.82	−0.4	8.13
	60 (2722)	20 (907)	20 (907)	0	80	89.82	−0.43	8.02
	60 (2722)	20 (907)	20 (907)	0	60	89.29	−0.41	8.09
3	60 (2722)	20 (907)	18 (817)	2 (91)	100	87.77	−0.54	7.04
	60 (2722)	20 (907)	18 (817)	2 (91)	80	87.84	−0.51	7.41
	60 (2722)	20 (907)	18 (817)	2 (91)	60	87.43	−0.55	7.61
4	76 (3447)	24 (1089)	0	0	100	88	−0.43	9.51
	76 (3447)	24 (1089)	0	0	80	88.27	−0.22	10.79
	76 (3447)	24 (1089)	0	0	60	88.27	−0.28	10.46
5	76 (3447)	12 (544)	12 (544)	0	100	88.65	−0.29	10.11
	76 (3447)	12 (544)	12 (544)	0	80	87.01	−0.53	8.46
	76 (3447)	12 (544)	12 (544)	0	60	87.71	−0.46	9.48
6	76 (3447)	12 (544)	10 (454)	2 (91)	100	87.32	−0.33	9.09
	76 (3447)	12 (544)	10 (454)	2 (91)	80	86.93	−0.44	9.09
	76 (3447)	12 (544)	10 (454)	2 (91)	60	87.16	−0.41	9.33
7	90 (4082)	10 (454)	0	0	100	87.26	−0.36	10.08
	90 (4082)	10 (454)	0	0	80	87.85	−0.19	10.83
	90 (4082)	10 (454)	0	0	60	87.86	−0.1	11.72
8	90 (4082)	5 (227)	5 (227)	0	100	87.24	−0.38	10.06
	90 (4082)	5 (227)	5 (227)	0	80	87.24	−0.33	10.53
	90 (4082)	5 (227)	5 (227)	0	60	87.3	−0.28	10.88
9	90 (4082)	5 (227)	3 (136)	2 (91)	100	86.13	−0.48	8.63
	90 (4082)	5 (227)	3 (136)	2 (91)	80	86.43	−0.41	9.53
	90 (4082)	5 (227)	3 (136)	2 (91)	60	86.33	−0.43	9.38
10	97.5 (4423)	0	0	2.5 (113)	100	85.41	−0.42	8.95
	97.5 (4423)	0	0	2.5 (113)	80	85.61	−0.4	10.14
	97.5 (4423)	0	0	2.5 (113)	60	85.7	−0.34	10.44

**Table 2:** Zeta Potentials of Tens Rolls of Nonwoven Fabrics Produced at 60 Bar. Note that Plateau Potential refers to ζ_plateau._

Sample	Weight (g/m^2^)	Pressure (bar)	Plateau Potential (mV)	∆ζ	R^2^	ζ_0_ (mV)	ζ_∞_ (mV)	Swell Ratio	IEP	k
1	33.8	60	−32	0.104	0.965	−41.35	−37.26	1.05	1.8	0.021
2	30.5	60	−33	0.070	0.873	−34.70	−33.50	1.02	1.6	0.084
3	25.8	60	−34	0.061	0.949	−40.02	−37.64	1.03	2.0	0.013
4	32.8	60	−30	0.130	0.947	−34.68	−30.14	1.07	1.6	0.009
5	26.5	60	−28	0.062	0.881	−31.32	−29.48	1.03	1.7	0.005
6	33.9	60	−29	0.042	0.791	−34.32	−32.57	1.03	1.6	0.026
7	31.2	60	−27.5	0.041	0.925	−27.40	−26.33	1.02	1.6	0.011
8	32.5	60	−30	0.085	0.752	−31.03	−29.64	1.02	1.6	0.001
9	33.9	60	−26	0.046	0.856	−26.56	−25.00	1.03	1.5	0.015
10	27.8	60	−25	0.047	0.904	−26.60	−25.45	1.02	1.6	0.015

**Table 3:** Details of the Resultant Tactile Descriptor Values.

Sample			SP	CR	FL	ST	SF	TFHV = SP + CR + FL + ST	SN *	FMR (* 1.0 × 10^−1^ mm^2^)
			(* 1.0 × 10^−4^ Joules)							
1	MD	Mean	20.16	6.34	8.52	36.03	45.18	71.09	0.90	1.68
		SD	4.15	1.66	2.19	6.22	8.19	11.55	0.27	0.16
	CD	Mean	17.25	3.77	6.38	28.25	35.25	55.65	1.71	2.12
		SD	1.30	0.18	0.53	1.37	1.89	2.73	0.02	0.18
	Whole fabric	Mean	18.71	5.05	7.45	32.14	40.21	63.37	1.31	2.71
		SD	1.65	0.68	0.90	2.53	3.38	11.40	0.10	0.12
4	MD	Mean	24.18	5.56	8.25	37.66	46.53	75.73	0.68	1.59
		SD	5.97	1.74	2.01	7.97	9.76	14.42	0.08	0.20
	CD	Mean	18.64	3.76	6.24	28.74	35.48	57.37	2.38	2.76
		SD	2.76	0.75	1.19	2.85	3.91	5.15	0.54	0.17
	Whole fabric	Mean	21.41	4.66	7.24	33.20	41.01	66.55	1.53	3.18
		SD	2.78	0.98	1.29	4.19	5.39	14.19	0.22	0.16
7	MD	Mean	19.40	5.59	7.67	32.99	41.18	65.69	1.00	1.48
		SD	0.79	0.41	0.28	0.89	0.81	0.93	0.11	0.22
	CD	Mean	14.24	4.39	5.70	25.26	31.38	49.59	4.17	2.79
		SD	3.23	0.76	1.13	4.14	5.35	7.43	3.69	0.38
	Whole fabric	Mean	16.82	4.99	6.68	29.13	36.28	57.64	2.59	3.16
		SD	0.99	0.40	0.57	1.52	2.05	9.64	1.49	0.32
6	MD	Mean	17.63	4.75	6.37	29.11	36.00	57.89	0.84	1.55
		SD	1.83	1.20	1.28	3.57	4.81	6.43	0.19	0.43
	CD	Mean	13.70	2.68	4.01	21.21	25.79	41.63	2.13	3.17
		SD	0.97	0.16	0.27	0.75	0.94	1.30	1.44	0.40
	Whole fabric	Mean	15.67	3.72	5.19	25.16	30.90	49.76	1.49	3.53
		SD	0.43	0.51	0.54	1.15	1.62	9.36	0.54	0.44
8	MD	Mean	16.42	5.26	5.98	28.72	35.25	56.41	0.97	1.39
		SD	0.91	1.33	1.00	3.19	4.02	5.25	0.28	0.24
	CD	Mean	13.64	3.86	4.70	23.64	28.84	45.85	3.80	2.56
		SD	1.49	0.47	0.36	1.37	1.79	2.42	1.67	0.13
	Whole fabric	Mean	15.03	4.56	5.34	26.18	32.05	51.13	2.38	2.92
		SD	0.83	0.35	0.53	1.64	2.13	6.68	0.79	0.05
9	MD	Mean	15.67	3.58	5.35	25.90	31.91	50.49	0.73	1.98
		SD	2.25	1.65	1.29	2.59	3.77	4.41	0.10	0.27
	CD	Mean	11.03	3.29	4.02	19.96	24.41	38.30	4.03	3.17
		SD	0.32	0.04	0.25	0.21	0.13	0.23	3.65	0.20
	Whole fabric	Mean	13.35	3.44	4.69	22.93	28.16	44.40	2.38	3.74
		SD	1.01	0.67	0.42	1.14	1.55	6.85	1.50	0.09

* Where fabric smoothness (SN) in machine direction (MD) is available for those aperture hydroentangled fabrics.

## References

[1] Condon B., Gary L., Sawhney A.P.S., Reynolds M., Slopek R., Delhom C.D., Hui D. (2010). Properties of nonwoven fabrics made with ultraclean cotton. World J. Eng..

[2] Sawhney P., Allen C., Reynolds M., Condon B., Slopek R. (2012). Effect of water pressure on absorbency of hydroentangled greige cotton non-woven fabrics. Text. Res. J..

[3] Umachitra G., Bhaarathidhurai (2012). Disposable baby diaper—A threat to the health and environment. J. Environ. Sci. Eng..

[4] Wakelyn P.J., Bertoniere N.R., French A.D., Thibodeaux D.P., Triplett B.A., Rousselle M.A., Goynes W.R., Edwards J.V., Hunter L., McAlister D.D. (2007). Cotton Fiber Chemistry and Technology.

[5] Campbell R.L. (1987). Clinical tests with improved disposable diapers. Pediatrician.

[6] Erasala G.N., Merlay I., Romain C. (2007). Evolution of disposable diapers and reduction of diaper dermatitis. Arch. Pediatr..

[7] Dey S., Kenneally D., Odio M., Hatzopoulos I. (2016). Modern diaper performance: Construction, materials, and safety review. Int. J. Dermatol..

[8] Stawski D., Bellmann C. (2009). Electrokinetic properties of polypropylene textile fabrics containing deposited layers of polyelectrolytes. Colloids Surf. A Physicochem. Eng. Asp..

[9] White C.F. The Wonderful World of Nonwovens. http://www.perinijournal.it/Items/en-US/Articoli/PJL-19/The-Wonderful-World-of-Nonwovens.

[10] Derler S., Gerhardt L.C. (2012). Tribology of skin: Review and analysis of experimental results for the friction coefficient of human skin. Tribol. Lett..

[11] Cottenden A.M., Cottenden D.J., Karavokiros S., Wong W.K. (2008). Development and experimental validation of a mathematical model for friction between fabrics and a volar forearm phantom. Proc. Inst. Mech. Eng. H.

[12] Gerhardt L.C., Mattle N., Schrade G.U., Spencer N.D., Derler S. (2008). Study of skin-fabric interactions of relevance to decubitus: Friction and contact-pressure measurements. Skin Res. Technol..

[13] Gerhardt L.C., Strassle V., Lenz A., Spencer N.D., Derler S. (2008). Influence of epidermal hydration on the friction of human skin against textiles. J. R. Soc. Interface.

[14] Pan N. (2007). Quantification and evaluation of human tactile sense towards fabrics. Int. J. Des. Nat..

[15] Yenket R., Chambers E., Gatewood Barbara M. (2007). Color has little effect on perception of fabric handfeel tactile properties in cotton fabrics. J. Sens. Stud..

[16] Mrs. Stewart’s Bluing—Why Bluing for Whatening Fabric?. http://mrsstewart.com/purpose-of-bluing/.

[17] Wan M., Zhou S., Jiao P., Cao C., Guo J. (2013). Synthesis, physical properties and cytotoxicity of stilbene-triazine derivatives containing amino acid groups as fluorescent whitening agents. J. Fluoresc..

[18] Kawabata S. (1980). The Standardization and Analysis of Hand Evaluation.

[19] Yaman N., Şenol F.M., Gurkan P. (2011). Applying artificial neural networks to total hand evaluation of disposable diapers. J. Eng. Fabr. Fibers.

[20] Brand R.H. (1964). Measurement of fabric aesthetics: Analysis of aesthetic components. Text. Res. J..

[21] Wool Handlemeter. http://www.woolcomfortandhandle.com/index.php/wool-handlemeter.

[22] Hu J. (2006). Characterization of Sensory Comfort of Apparel Products.

[23] Mao N. Towards objective discrimination & evaluation of fabric tactile properties: Quantification of biaxial fabric deformations by using energy methods. Proceedings of the 14th AUTEX World Textile Conference.

[24] Kamalha E., Zeng Y., Mwasiagi J., Kyatuheire S. (2013). The Comfort Dimension; A Review of Perception in Clothing.

[25] Mao N., Taylor M. (2012). Evaluation Apparatus and Method.

[26] Edwards V.J., Mao N., Russell S., Carus E., Condon B., Hinchliffe D., Gary L., Graves E., Bopp A., Wang Y. (2015). Fluid handling and fabric handle profiles of hydroentangled greige cotton and spunbond polypropylene nonwoven topsheets. Proc. Inst. Mech. Eng. Part L J. Mater. Des. Appl..

[27] Easson M.W., Condon B.D., Reynolds M.L., Franqui R., Bland J. (2017). Non-bleaching heather method for improved whiteness of greige cotton. J. Eng. Fabr. Fibers.

[28] Grahame D.C. (1947). The electrical double layer and the theory of electrocapillarity. Chem. Rev..

[29] Helmholtz H. (1879). Studien über electrische grenzschichten. Ann. Phys..

[30] Fairchild M. (2013). Color Appearance Models.

[31] Edwards J.V., Prevost N., Condon B., Batiste S., Reynolds M., Allen C., Ducruet M., Sawhney P., Parikh D., Slopek R. (2012). Electrokinetic properties of functional layers in absorbent incontinence nonwoven products. Text. Res. J..

[32] Ribitsch V., Stana-Kleinscheck K. (1998). Characterizing textile fiber surfaces with streaming potential measurements. Text. Res. J..

[33] Edwards V., Condon B., Sawhney P., Reynolds M., Allen C., Nam S., Bopp A., Chen J., Prevost N. (2013). Electrokinetic analysis of hydroentangled greige cotton–synthetic fiber blends for absorbent technologies. Text. Res. J..

[34] Kim H.S., Pourdeyhimi B., Desai P., Abhiraman A.S. (2001). Anisotropy in the mechanical properties of thermally spot-bonded nonwovens: Experimental observations. Text. Res. J..

[35] Klevaityte R., Masteikaite V. (2008). Anisotropy of woven fabric deformation after stretching. Fibres Text. Eastern Eur..

[36] Richer C. (2011). Trends in disposable diaper design. Nonwovens Ind..

[37] Gupta B.S. Fluid imbibitions behavior of nonwovens containing cellulose acetate and it blends with synthetic and other cellulosic fibers. Proceedings of the Beltwide Cotton Conferences.

[38] Gupta B.S., Hong C.J. (1994). Changes in web dimensions during fluid uptake and the impact of absorbency. Tappi J..

